# Detection of loci exhibiting pleiotropic effects on body weight and egg number in female broilers

**DOI:** 10.1038/s41598-021-86817-8

**Published:** 2021-04-02

**Authors:** Eirini Tarsani, Andreas Kranis, Gerasimos Maniatis, Ariadne L. Hager-Theodorides, Antonios Kominakis

**Affiliations:** 1 Department of Animal Science and Aquaculture, Agricultural University of Athens, Iera Odos 75, 11855 Athens, Greece; 2 Aviagen, Newbridge, EH28 8SZ Midlothian UK; 3 The Roslin Institute, University of Edinburgh, Midlothian, EH25 9RG UK

**Keywords:** Genome-wide association studies, Genomics

## Abstract

The objective of the present study was to discover the genetic variants, functional candidate genes, biological processes and molecular functions underlying the negative genetic correlation observed between body weight (BW) and egg number (EN) traits in female broilers. To this end, first a bivariate genome-wide association and second stepwise conditional-joint analyses were performed using 2586 female broilers and 240 k autosomal SNPs. The aforementioned analyses resulted in a total number of 49 independent cross-phenotype (CP) significant SNPs with 35 independent markers showing antagonistic action i.e., positive effects on one trait and negative effects on the other trait. A number of 33 independent CP SNPs were located within 26 and 14 protein coding and long non-coding RNA genes, respectively. Furthermore, 26 independent markers were situated within 44 reported QTLs, most of them related to growth traits. Investigation of the functional role of protein coding genes via pathway and gene ontology analyses highlighted four candidates (*CPEB3, ACVR1, MAST2* and *CACNA1H*) as most plausible pleiotropic genes for the traits under study. Three candidates (*CPEB3, MAST2* and *CACNA1H*) were associated with antagonistic pleiotropy, while *ACVR1* with synergistic pleiotropic action. Current results provide a novel insight into the biological mechanism of the genetic trade-off between growth and reproduction, in broilers.

## Introduction

Reproductive traits in livestock species often show an antagonistic relationship with growth traits that is manifested as negative genetic correlation between single members of trait complexes. In chicken, a typical example is the negative genetic correlation (r_g_) estimated between body weight (BW) and egg number (EN) with r_g_ estimates in the range from − 0.05 to − 0.55^1–3^. In general, the most important source for genetic correlations is usually pleiotropy^4^, however, genetic correlations may also arise from linkage disequilibrium (LD) among distinct loci^5^.

One possible way of identifying plausible pleiotropic genetic loci is to perform multivariate or univariate GWAS of traits under interest. The resulting marker trait(s) associations are termed cross-phenotype (CP) associations^6^. While multivariate approaches^7^ allow for direct identification of CP associations, in the context of univariate analyses, detection of CP associations relies on aggregating results of single traits analyses via meta-analysis techniques^8^.

When searching for pleiotropic loci via GWAS, it is important to bear in mind that CP associations are based on statistical evidence regardless of the underlying cause^9^ and are not necessarily indicative of pleiotropic genetic variants.

On the contrary, pleiotropy occurs when a genetic locus truly affects more than one trait, simultaneously. When beneficial effects of a genetic factor on one trait are accompanied by negative effects on the other trait, antagonistic pleiotropy (AP) exists^10,11^. In contrast to AP, synergistic pleiotropy^12,13^ (SP) occurs when a genetic variant simultaneously either increases or decreases performance in two different traits.

Based on the mechanisms of action, pleiotropy can be distinguished in: biological (or horizontal), mediated (or vertical) and spurious pleiotropy^6,14^. Specifically, in biological pleiotropy, a genetic variant or a gene affects multiple phenotypes since causal variants for different phenotypes can be colocalized in the same gene. In mediated pleiotropy, there is a causal relationship between two phenotypes so as a variant exerts an effect on one phenotype through the another one while spurious pleiotropy refers to a falsely association between marker and phenotypes due to bias, misclassification or linkage disequilibrium (LD). To overcome the challenge of spurious pleiotropy in the latter case, approaches such as LD pruning and conditional and joint analysis (cojo) can be applied to alleviate the high SNP interdependency arising from LD and to select the LD independent SNPs.

In chicken, CP associations have already been reported by GWAS for various traits such as daily feed intake and efficiency^15^ and for egg weights at different ages^16,17^. Nevertheless, no GWAS has, so far, been reported with the aim to discover genetic variants associated with body weight (BW) and egg number (EN) in chickens.

Driven from the scarcity of relevant reports, we have elaborated the present study with the aim to identify genetic variants and genes simultaneously affecting BW and EN in chickens. To this end, first we conducted a bivariate GWAS to identify SNP signals associated with both traits. As LD could generate spurious pleiotropic associations (see reviews^6,14^) we then applied conditional and joint analysis (cojo) of the SNP signals obtained from bivariate analysis to identify LD-independent CP SNPs. Finally, we investigated the functional role of the candidate genes underlying the independent CP SNPs in attempts to propose the most relevant pleiotropic genes implicated in the genetic control of traits under study.

## Results

### Comparison of genome-wide significant SNPs found by bivariate analysis, BW univariate analysis and EN univariate analysis

**Figure 1:**
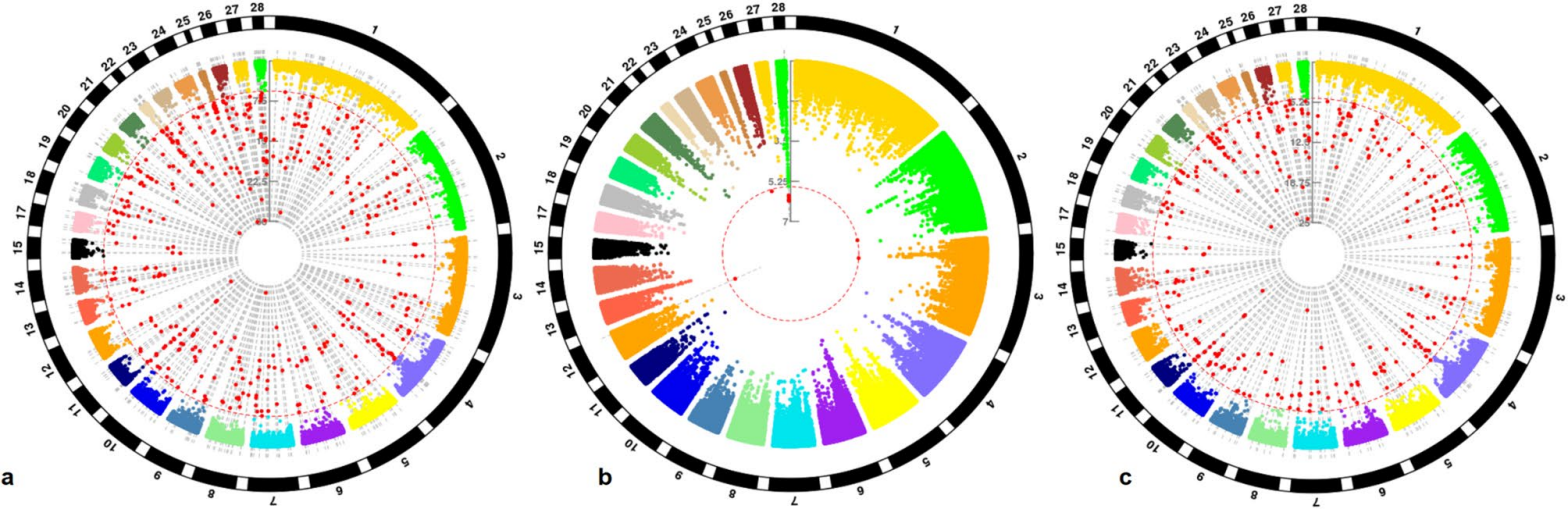
Circular Manhattan plots showing the − log_10_(p values) of SNPs across the 28 autosomal chromosomes for body weight (BW) (**a**), egg number (EN) (**b**) and both traits (**c**), respectively. Red dots in Manhattan plots denote the genome-wide significant SNPs. Plots were constructed using the CMplot package (https://github.com/YinLiLin/R-CMplot) in R (http://www.r-project.org/).

**Figure 2:**
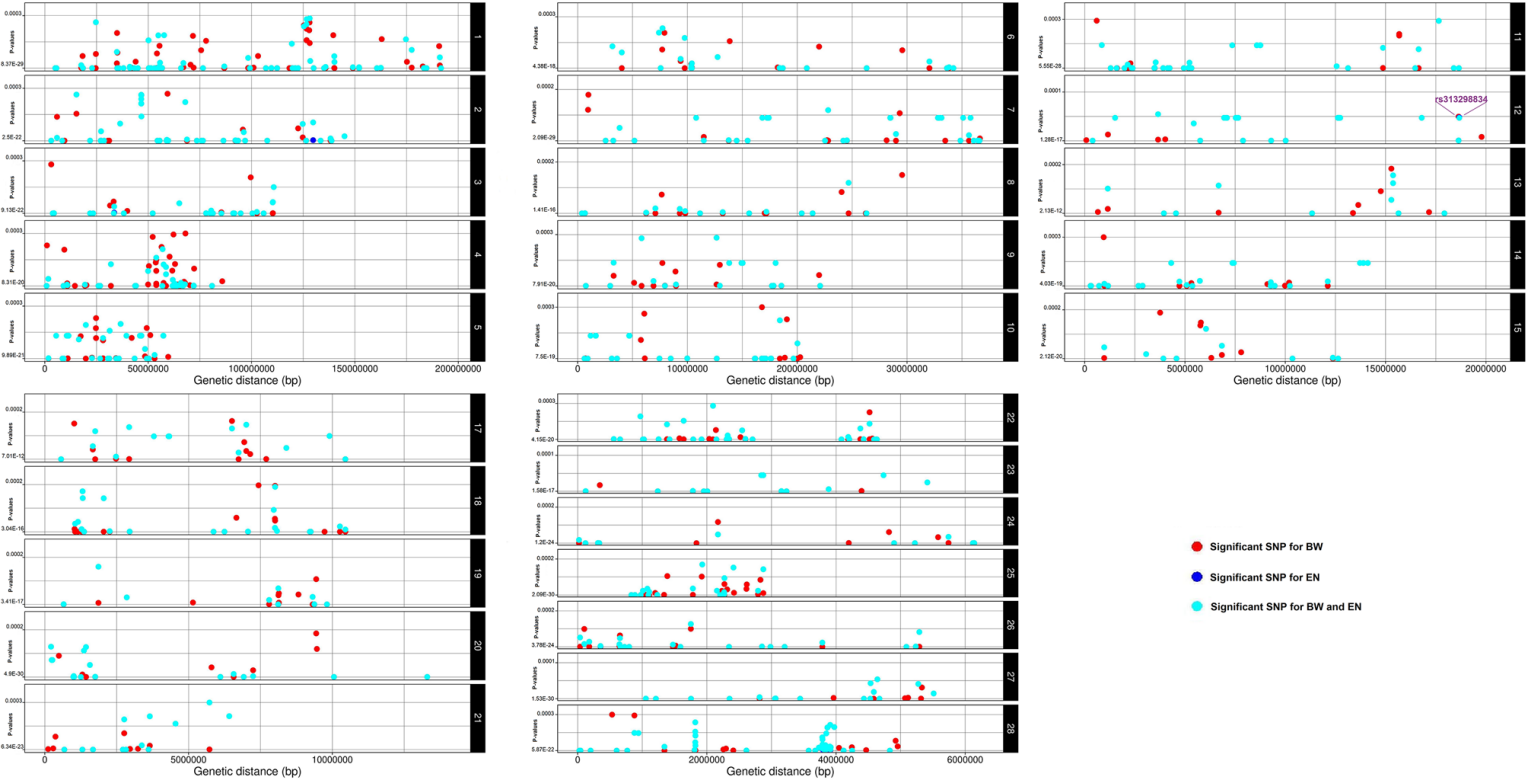
Graphic depiction showing the positions of the genome-wide significant SNPs (denoted as points) and their p values from univariate and bivariate analyses aligned along each autosome. Black labels with number denote the chromosomes. Each SNP position is linked to at least one colored circle representing the associated trait (s) (i.e. body weight (BW): red circle, egg number (EN): blue circle and both traits: cyan circle). The common SNP between the three analyses (2 univariate and 1 bivariate analyses) is presented with purple color. Plot was constructed using the shinyChromosome (https://yimingyu.shinyapps.io/shinyChromosome^59^).

**Figure 3:**
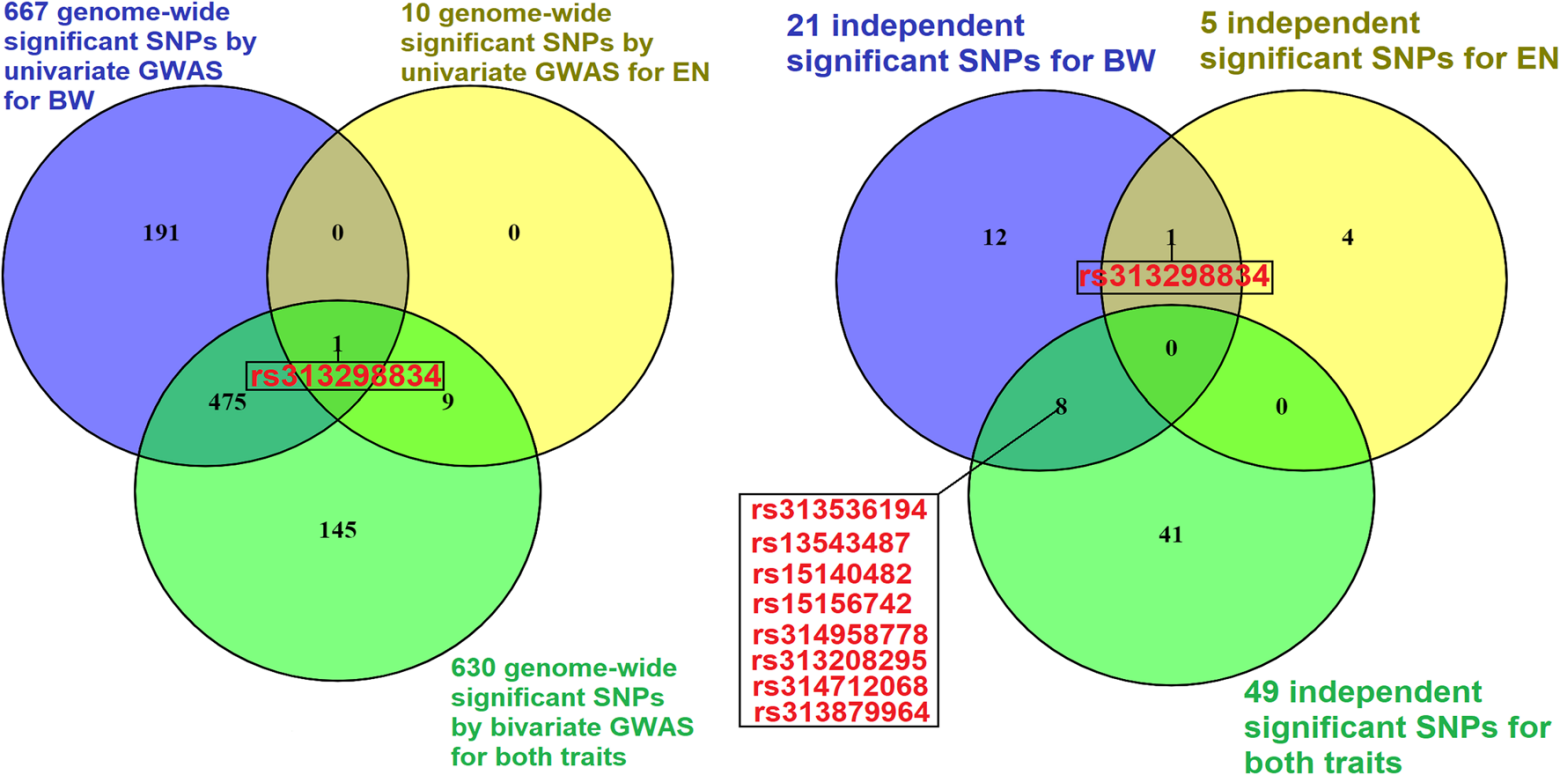
Venn diagrams showing the number of common significant SNPs between analyses. The left Venn diagram presents the number of common significant SNPs between bivariate association analysis (both traits: green color) and univariate association analyses (body weight (BW): blue color and egg number (EN): yellow color) while the right Venn diagram presents the number of common significant SNPs after conditional and joint (cojo) analyses. Some common SNPs (with their rsids) are also given with red color. Venn diagrams were constructed by VENNY 2.1^60^.

Estimations of the genomic inflation factors (univariate analyses: *λ*_*BW*_ = 0.86, *λ*_*ΕΝ*_ = 0.95, bivariate analysis: *λ* = 0.85) were less than 1 indicating the absence of population structure or artifacts in the present data. Furthermore, the genomic genetic correlation (r_g_) between the two traits was estimated as high as − 0.183 ± 0.15 (results not shown). Figure 1 shows the profiles of the SNP p values (expressed as − log_10_ values) across the three GWAS. Specifically, a total number of 667 genome-wide significant SNPs (FDR p value < 0.10) were detected by BW univariate analysis and these SNPs were dispersed across the 28 autosomes (Figs. 2, 3). For EN univariate analysis, a total number of 10 SNPs across five autosomes (2, 3, 12, 26 and 28) were found to reach genome-wide significance (FDR p value < 0.10) (Figs. 2, 3). The bivariate analysis resulted in 630 genome-wide significant (FDR p value < 0.10) CP SNPs across the 28 autosomes (Figs. 2, 3).

Comparison of SNP signals across the three association analyses resulted in one common significant marker (*rs313298834*) on GGA12 (Figs. 2, 3). In addition, a total number of 475 SNPs were common between the BW univariate analysis and the bivariate analysis and all significant SNPs (n = 10) for EN were also detected by bivariate analysis (Fig. 3).

### LD-independent significant SNPs

Cojo-GCTA analyses resulted in a total number of 49 independent CP SNPs while 21 and 5 independent significant SNPs were detected for BW and EN, respectively (Fig. 3, Supplementary Table S1). As observed in Fig. 3, no independent SNP was common between the three analyses. Nevertheless, markers were common between the BW univariate analysis and the bivariate analysis (Fig. 3).

Table 1 presents a detailed description of the 49 independent CP associations across the 25 autosomes (1–15, 17–21 and 23–28). The maximum number (n = 9) of independent markers were located on GGA1 while marker *rs315329074* (GGA27) presented the lowest p value (6.13E-32) after ‘cojo’ analysis. Table 1 along with Supplementary Fig. S1 also shows the estimated marker effect sizes (*β*) on the two traits obtained from bivariate analysis. In accordance with antagonistic pleiotropic action i.e. positive effects on one trait and negative effects on the other trait, the estimated effect sizes for the majority (35 out of 49) of the CP associations displayed opposing signs (Supplementary Fig. S1).

**Table 1:** Independent cross-phenotype (CP) significant SNPs for body weight and egg number in broilers.n

SNP ID	GGA	Position (bp)^a^	*β*_*BW*_	*β*_*EN*_	P value from bivariate analysis	FDR p value	P value from COJO^b^
*rs315275636*	1	6206137	5.655	− 1.212	7.91E−12	2.70E−08	1.17E−12
*rs317275973*		23082139	− 4.514	2.223	1.82E−14	1.62E−10	1.29E−17
*rs312392044*		35990963	5.732	− 0.566	1.41E−13	8.96E−10	2.71E−14
*rs316780156*		87938572	3.453	− 0.367	8.75E−06	0.005942	2.93E−07
*rs315007062*		100596308	3.556	0.737	3.22E−09	4.86E−06	1.74E−09
*rs315995534*		110869322	2.073	− 2.200	8.56E−07	0.000739	2.13E−06
*rs317073055*		121235094	5.485	− 0.913	4.67E−13	2.39E−09	7.06E−14
*rs317590244*		136269771	4.125	− 0.769	2.57E−11	7.88E−08	3.78E−12
*rs316472061*		185926511	5.680	− 0.613	1.10E−13	7.39E−10	1.95E−14
*rs14135719*	2	8489508	5.556	− 0.980	2.87E−11	8.57E−08	4.41E−12
*rs13543487*		27103453	3.484	0.347	1.92E−13	1.16E−09	5.89E−14
*rs317979230*		59469333	4.052	0.222	1.19E−14	1.12E−10	3.06E−15
*rs315191969*		75552665	2.426	− 0.309	2.04E−08	2.49E−05	8.15E−08
*rs15140482*		107231066	4.770	− 1.392	2.23E−11	7.01E−08	3.28E−12
*rs15156742*		133305723	4.581	0.160	1.42E−15	1.96E−11	3.03E−16
*rs313125064*	3	21986853	3.289	− 2.777	2.93E−07	0.000271	2.13E−13
*rs317668107*		33354124	− 3.193	0.089	1.45E−11	4.81E−08	2.22E−17
*rs314958778*		52121842	3.481	1.330	1.58E−08	2.01E−05	2.04E−08
*rs313973628*	4	8970286	− 3.035	0.604	1.48E−10	3.46E−07	1.88E−08
*rs313178030*		26530662	5.752	− 1.122	2.27E−13	1.34E−09	3.38E−14
*rs317953448*		43384266	4.267	− 0.882	7.08E−12	2.46E−08	1.02E−12
*rs15608447*		66459916	− 2.582	− 1.263	9.09E−09	1.24E−05	3.66E−10
*rs313208295*		80292623	5.069	− 1.164	4.60E−11	1.31E−07	6.62E−12
*rs312798022*	5	8828819	5.396	− 0.853	3.11E−12	1.26E−08	4.08E−13
*rs313257959*		30658287	5.614	− 0.332	7.37E−14	5.11E−10	1.39E−14
*rs314038572*		50471323	4.876	− 0.842	5.00E−10	9.73E−07	7.49E−11
*rs314529054*	6	21832302	2.039	− 0.633	1.78E−07	0.000172	3.12E−07
*rs314712068*		35135780	3.939	− 0.485	2.74E−10	5.93E−07	2.44E−10
*rs313879964*	7	36286374	6.084	0.215	4.11E−22	2.42E−17	2.15E−22
*rs314425715*	8	770143	4.218	− 2.068	1.21E−08	1.59E−05	3.17E−09
*rs317902708*		21684030	4.932	− 0.771	1.30E−10	3.15E−07	3.88E−10
*rs317315660*	9	17942760	− 3.302	1.039	9.72E−07	0.000818	4.68E−12
*rs14952656*	10	17996013	5.234	− 0.789	1.62E−10	3.67E−07	1.08E−29
*rs316546378*	11	5124955	2.849	0.314	2.87E−05	0.016969	4.87E−07
*rs318098582*		18407493	5.686	− 0.633	2.66E−21	1.05E−16	5.21E−22
*rs318048363*	12	6154483	5.078	− 0.824	1.09E−10	2.75E−07	1.63E−11
*rs318032338*	13	16259361	− 2.251	− 1.391	7.64E−09	1.07E−05	6.22E−07
*rs317631529*	14	5738298	− 3.141	0.735	1.86E−14	1.62E−10	1.16E−11
*rs314778226*	15	4845973	− 4.360	0.131	3.27E−14	2.57E−10	4.93E−15
*rs317370260*	17	1629390	2.168	0.418	0.000148	0.061232	2.89E−08
*rs313997974*	18	6177837	5.012	− 1.160	9.66E−10	1.74E−06	5.29E−13
*rs313536194*	19	9937564	5.266	− 1.273	1.66E−10	3.73E−07	4.10E−15
*rs317414603*	20	6729013	4.271	− 0.693	4.47E−23	3.51E−18	7.37E−24
*rs314420361*	21	698421	5.308	0.203	1.31E−15	1.96E−11	3.75E−16
*rs317101069*	23	3379059	2.747	1.986	2.23E−15	2.77E−11	4.59E−09
*rs14291881*	24	150829	3.875	− 1.075	2.59E−19	7.63E−15	4.07E−20
*rs316343530*	26	2854350	− 2.915	− 1.896	2.24E−06	0.001699	7.48E−13
*rs315329074*	27	6920352	− 4.810	1.438	4.23E−25	9.97E−20	6.13E−32
*rs314496246*	28	3661043	4.730	0.156	1.42E−15	1.96E−11	1.98E−11

The marker effect sizes (*β*_*BW*_*, β*_*EN*_) of CP-SNPs on the two traits are also provided.

^a^Positions are based on GRCg6a assembly.

^b^COJO stands for conditional and joint analysis.

Note that the estimated genomic r_g_ (− 0.183) is a weighted average of effect sizes of markers exhibiting antagonistic, synergistic and non pleiotropic action and only in the extreme case of r_g_ = − 1 all the implicated markers would exhibit opposing effects on both traits.

### Effect prediction of the independent CP significant SNPs and identification of positional candidate genes and published QTLs

A total number of 40 positional candidate genes (of which 24 were annotated genes) were identified as lying within 33 independent SNPs while 16 SNPs were intergenic variants (Supplementary Table S2). Specifically, 33 SNPs were located within 26 protein coding genes and 14 long non-coding (lnc) RNA genes (Supplementary Table S2). Of these SNPs, one was a missense variant of gene *ZC3H18*, one a synonymous variant of gene *EXTL1* and 23 were intron variants of annotated genes (*CELF2, PTPRZ1, PTPRB, EIF1AX, NOL4, TMEM206, SLAIN2, SORCS2, FMN1, MARK3, CPEB3, EBF3, ACVR1, AMY2A, MAST2, TBL1XR1, CHSY1, CACNA1H, NPHP4, VPS11, PLXNA2* and *CACNB1*).

**Figure 4:**
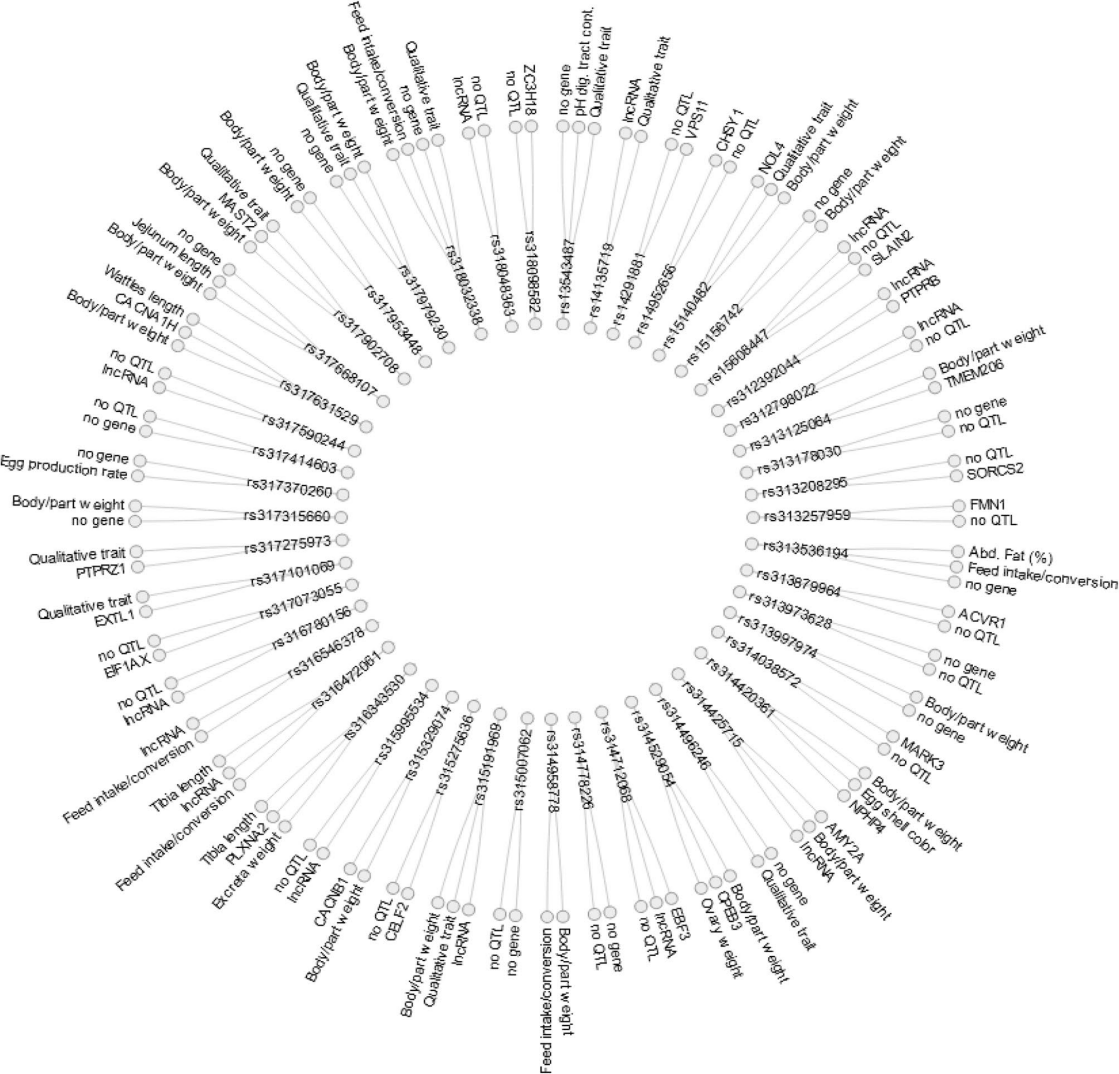
Radial network showing the independent cross-phenotype significant SNPs (with *rsids*) and the corresponding positional candidate genes and/or published QTLs in the searched regions. Note that QTLs were grouped according to their trait relevance. Figure was constructed using data.tree and networkD3 R packages.

Figure 4 provides a view for the independent CP significant SNPs (n = 49) and the corresponding positional candidate gene(s) and published QTL(s). With regard to QTLs, 26 independent SNPs lied within 44 previously reported QTLs (Supplementary Table S2). As seen in Fig. 4, the majority of QTLs were related to body weight (hatch, at 21 or 36 days) or body parts weight (e.g. femur weight, proventriculus weight, breast muscle weight, ileum weight, wattles and comb weight), followed by QTLs related to Qualitative Traits (e.g. feathered feet, feather colour extended black and feather crested head) and finally QTLs related to feed, dry matter intake or feed conversion. Notably, one of the independent SNPs i.e. *rs314529054* was located within a region of GGA6 where an ovary weight QTL and a body weight QTL are reported. Marker *rs314529054* also lied within gene *CPEB3* (Supplementary Table S2). Finally, *rs317370260* lied within a region of GGA17 where a QTL related to egg production rate is reported. No positional gene was present in the area with only a lncRNA lying in proximity (distanced 5322 bp) to the marker.

**Figure 5:**
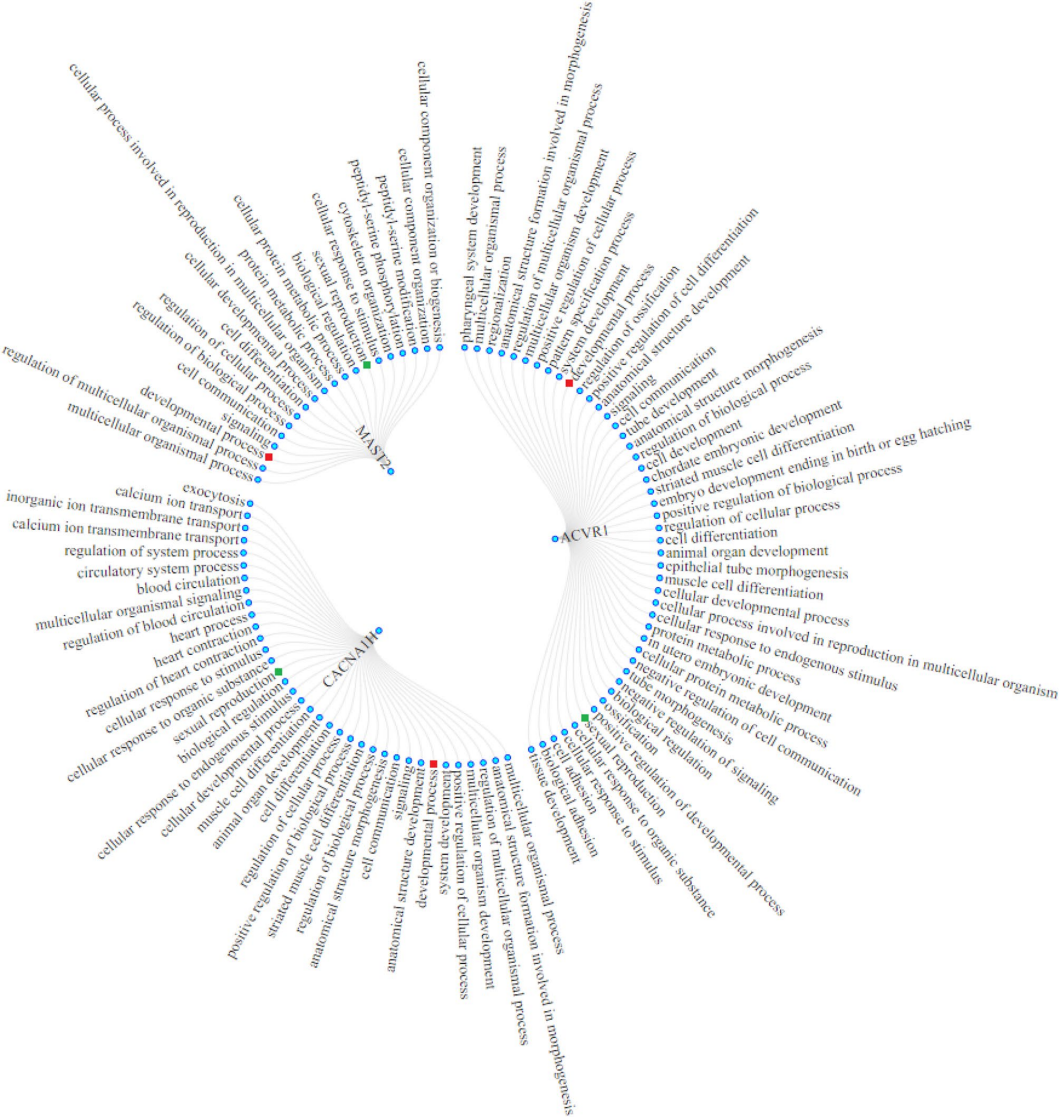
Radial network displaying the *ACVR1, MAST2* and *CACNA1H* genes and their respective biological processes. Red color denotes the developmental process while reproduction is marked with green color. Figure was constructed using data.tree and networkD3 R packages.

### Bioinformatics analyses

All annotated chicken genes were recognized by GeneCodis 4.0. MEA revealed 15 genes participating in significantly enriched concurrent GO BP and/or pathway annotations (Supplementary Table S3). Furthermore, the LAGO tool recognized all but two (*NOL4* and *ZC3H18*) candidate genes and highlighted a total number of 120 enriched GO BPs (Supplementary Table S4).

Several candidate genes displayed growth functional relevance (e.g. GO:0032502: developmental process: participating genes *ACVR1, CACNA1H, CHSY1, CPEB3, EBF3, EXTL1, FMN1, MAST2, NPHP4, PLXNA2, PTPRB, PTPRZ1, TBL1XR1ACVR1, PLXNA2, ACVR1, CACNA1H, CHSY1, CPEB3, EBF3, EXTL1, FMN1, NPHP4, PLXNA2, PTPRB, PTPRZ1, TBL1XR1*). Nevertheless, only *CPEB3, ACVR1*, *CACNA1H* and *MAST2* were found to participate in reproduction related processes. Specifically, *CPEB3* was associated with three concurrent annotations related to reproduction (oocyte meiosis, regulation of translation and progesterone-mediated oocyte maturation, FDR p value = 0.01) (Supplementary Table S3). Jointly, the rest three genes (*ACVR1*, *CACNA1H* and *MAST2*) were associated with sexual reproduction (GO:0019953) while *ACVR1* and *MAST2* were further associated with *cellular process involved in reproduction in multicellular organism* (GO:0022412) (Supplementary Table S4; Fig. 5).

Of the four above genes, *ACVR1* displayed the highest number of significant concurrent annotations (n = 55 GO BPs) and was also member of two pathways: *positive regulation of BMP signaling* (GO:0030513) and *TGF-beta signaling pathway* (gga04350). Based on functional relevance derived from ontological and functional analyses, the four above genes (*ACVR1, CPEB3, MAST2* and *CACNA1H*) were nominated as most plausible pleiotropic candidate genes for BW and EN. The four above mentioned genes lied within respective numbers of SNPs i.e. *CPEB3* (*rs314529054,* (GGA6), *p*_*cojo*_ = 3.12E−07), *ACVR1 (rs313879964* (GGA7), *p*_*cojo*_ = 2.15E−22), MAST2 (*rs317902708* (GGA8), *p*_*cojo*_ = 3.88E−10) and *CACNA1H (rs317631529*, (GGA14), *p*_*cojo*_ = 1.16E−11) (Table 1). Estimated effect sizes (*β*) obtained from bivariate analysis for the respective markers are indicative of antagonistic pleiotropic action for the three genes i.e*. CPEB3 (β*_*BW*_ = 2.04, *β*_*EN*_ = − 0.63), *MAST2 (β*_*BW*_ = 4.93, *β*_*EN*_, =  − 0.77) and *CACNA1H (β*_*BW*_ = − 3.14, *β*_*EN*_ = 0.74) and synergistic action for *ACVR1 (β*_*BW*_ = 6.08, *β*_*EN*_ = 0.26, Table 1). Of the three genes exhibiting antagonistic action, *CPEB3* and *MAST2* affected positively and negatively BW and EN, respectively, while a reverse trend was observed for *CACNA1H*.

With regard to molecular function, the four candidate genes showed GO slim terms of binding (GO:0005488) and protein binding (GO:0005515), *ACVR1* and *MAST2* of kinase activity (GO:0016301), catalytic activity (GO:0003824) and transferase activity (GO:0016740), *CACNA1H* of ion transmembrane transporter activity (GO:0015075), channel activity (GO:0015267) and transporter activity (GO:0005215) and finally *CPEB3* of nucleic acid binding (GO:0003676) and translation regulator activity (GO:0045182) (results not shown).

## Discussion

In the present study, marker trait(s) association analyses along with in silico exploration of the biological role(s) of the implicated genes were jointly applied to refine our understanding of the genetic trade-off between BW and EN, in broilers. The genetic antagonism between the two traits was confirmed at the genome-wide level, as the estimate of the genomic genetic correlation between BW and EN was as high as − 0.18 herein, in concordance with previous findings^1–3^. As this genome-wide estimate only describes the cumulative CP effects of all implicated causal loci, in this study we further attempted to identify genetic variants with strong statistical associations for both traits, quantify their patterns of pleiotropic effects and explore the involved biological processes and/or pathways.

Marker trait(s) association analysis verified previous results, by revealing one common SNP (*rs313298834*) on GGA12 that has been associated with EN in female broilers^18^ and five more markers (*rs317668107* on GGA3, *rs15608447* on GGA4, *rs318098582* on GGA11, *rs317414603* on GGA20 and *rs315329074* on GGA27) associated with BW^19^ in broilers. We also noted a higher number of SNP signals passing the genome-wide FDR significance threshold (set up to 0.10 here) for BW when compared to EN. As the yield of GWAS critically depend on the underlying effect-size distribution of the implicated variants^20^, this is not a surprising finding and it may be attributed to the lower heritability estimate for EN (0.17) when contrasted to the respective estimate (0.30) for BW.

Bivariate analysis identified a vast number of genome-wide significant CP SNPs. To ensure that CP associations did not arise from LD between markers (spurious pleiotropy^6^), a critical step in the present study was to identify only LD-independent SNPs via stepwise cojo analysis, as in other GWAS^21,22^. After overcoming the LD challenge, our results disclosed several genetic variants simultaneously affecting BW and EN with some of them pointing to most promising pleiotropic genes, as we discuss below.

The first SNP that served as a proxy to a plausible pleiotropic gene was *rs314529054* (GGA6). The marker is located within two reported QTLs related to ovary and body weight and lies within *CPEB3* (*cytoplasmic polyadenylation element binding protein 3*) gene. Ontological and functional analysis suggested that the gene in question may be considered as a true pleiotropic gene, however, literature evidence on its functional relevance to the traits examined here is shortcoming. In mice, *CPEB* has been reported to control polyadenylation and translation during the dictyate stage of oocyte development and this regulation has also a profound influence on folliculogenesis^23^.

In contrast to the previous marker, *rs313879964* (GGA7) pointed at a gene with a highly likely pleiotropic function. This specific marker lied within *ACVR1* (*serine/threonine-protein kinase receptor or activin receptor type I or activin a receptor, type 1,* also known as *ALK2*) gene. This gene participates in several growth and reproduction related GO BPs and has well documented involvement in biological phenomena such as those examined here. As MEA showed, *ACVR1* encodes for a bone morphogenetic protein (BMP) type I receptor of the transforming growth factor-beta (TGF-β) superfamily which plays a key role in cell growth while regulates several reproductive processes (such as follicular development and ovulation)^24^. In addition, *ACVR1* regulates reproduction via the BMP and anti-Müllerian hormone (AMH) signaling^25^. In chickens, AMH is required for the urogenital development and germ cell migration^26^, is presented in early development of follicles and is expressed in small follicles^27^. So far, the chicken *ACVR1* gene has been suggested as a positional candidate gene for body weight^28^, has a regulatory role during skeletal development in osteogenesis and chondrogenesis^29^ and is expressed within the chicken granulosa and thecal layers during ovarian follicle development^30^.

Two more markers, *rs317902708* (GGA8) and *rs317631529* (GGA14) pointed at two most promising pleiotropic genes i.e., *MAST2* and *CACNA1H*, respectively. Specifically, *MAST2* (*microtubule associated serine/threonine kinase 2*) has been previously detected by RNA-seq in visceral fat of broiler and layer females at the onset of sexual maturation^31^. Furthermore, human *MAST2* gene has been reported to be involved in PI3K-AKT signaling pathway^32^ that regulates various cellular processes, such as proliferation, growth, apoptosis and cytoskeletal rearrangement^33^. On the other hand, *CACNA1H (calcium voltage-gated channel subunit alpha1 H,* also known as *Cav3.2*) encodes for Cav3.2 channel that is a member of the voltage-gated calcium channel family. This gene participates in the T-type Ca^2+^ channels which contribute to signal transduction pathways regulating protein synthesis, development, proliferation and cell differentiation^34^ that are mainly expressed during embryonic development^34^. Particularly, these channels are involved in the early stages of muscle differentiation in humans^35^ and mice^36^. Female Cav3.2^-/-^ null mutant mice presented decreased body weight^37^ and reduced litter size^38^. Moreover, Cav3.2 may have a role in reproduction since it facilitates the influx of Ca^2+^ in mouse oocytes and eggs to maintain Ca^2+^ homeostasis during oocyte maturation and *post* fertilization^38^. The murine *Cacna1h* gene is also upregulated in the proestrus of the Gonadotropin-releasing hormone (GnRH) neurons^39^. GnRH determines the pattern of secretion of follicle stimulating hormone (FSH) and luteinising hormone (LH) that regulate the endocrine function and gamete maturation of gonads^40^. So far, the chicken *CACNA1H* gene has only been associated with egg quality^41^ and body weight^42^.

Based on the approach followed herein, three candidates (*CPEB3, MAST2* and *CACNA1H*) were identified as ‘trade-off’ genes i.e. exhibiting antagonistic pleiotropy, while *ACVR1* displayed synergistic pleiotropic action.

We hypothesize that the above four genes are indicative of horizontal pleiotropy although we acknowledge the scepticism of Jordan et al.^43^ who hypothesized that the pervasive horizontal pleiotropy observed in polygenic traits is, on some level, a logical consequence of widespread polygenicity. Present results seem to fairly support such a hypothesis as hundreds of markers with little individual effects on the traits could be detected in the present study, especially for BW.

Another interesting finding obtained herein was the presence of independent CP SNPs within long non-coding genes (lncRNAs: RNA transcripts greater than 200 bp in length). In nucleus, lncRNAs have been reported to function in-cis and in-trans whereby in-cis acting lncRNAs influence the expression of nearby genes^44^. Furthermore, lncRNAs can encode short peptides^44,45^ and function as molecular decoy for proteins or sponges for other transcripts (such as miRNAs)^44^. They can also regulate numerous functions such as epigenetic modification, transcription and post-transcription while playing a key role in tissue development, muscle contraction/relaxation^44^ and myogenesis^45^. In chickens, lncRNAs have been reported to regulate muscle development, lipid metabolism, egg production and disease resistance^46^.

To conclude, present results provide a novel insight in the genetic mechanism underlying antagonistic interplay between growth and reproduction in broilers. Further follow-up studies (e.g. fine mapping and gene expression studies) are warranted to experimentally verify present findings.

## Methods

### Data and quality control

Genotypic and phenotypic records were provided by Aviagen. The available data consisted of 2992 female broilers from a grand-grandparent (GGP) commercial line with phenotypic records on body weight (BW) at 35 days of age (average = 1822.7 g, SD = 143.6 g) and number of eggs (EN) per hen collected from 28 to 50 weeks of age (average = 132.4 eggs, SD = 29.8 eggs). Animals were genotyped using the 600 k Affymetrix HD SNP array^47^ resulting in a total number of 544,927 autosomal SNPs. Quality control (QC) was performed first at a sample and second at a marker level. At a sample level, 406 animals were excluded due to call rate < 0.99 and autosomal heterozygosity outside the 1.5 IQR (inter-quartile range: 0.013). At the marker level, 305,660 SNPs autosomal SNPs were excluded due to: call rate < 0.95, minor allele frequency (MAF) < 0.05 and LD pruning (r^2^ > 0.99 within windows of 1 Mb inter-marker distances). Finally, a total of 2586 samples and 239,267 autosomal SNPs were retained for further analyses. All QC criteria were applied using the SNP & Variation Suite software (http://www.goldenhelix.com).

### Univariate and bivariate association analyses

First, we performed univariate analyses to detect significant SNP associations for individual traits. The following univariate linear mixed model was applied:

$${\text{y}} = {\mathbf{W}}\alpha + {\text{x}}\beta + {\text{u}} + {\text{e}}$$

where *y* is a n × 1 vector of phenotypic values of BW or EN for n = 2586 animals, **W** is a n × 53 matrix of covariates of fixed effects including hatch (36 classes) and mating group (17 classes), *α* is a c × 1 vector of the corresponding coefficients, *x* is a n × 1 vector of marker genotypes (coded as 0, 1, and 2 according to the number of copies of the minor allele), *β* is the effect size of marker on BW or EN, *u* is a vector of random polygenic effects and *e* is a vector of random residuals. The random effects were assumed to be normally distributed with zero means and the following covariance structure:

$$Var\left[ {\begin{array}{*{20}c} u \\ e \\ \end{array} } \right] = \left[ {\begin{array}{*{20}c} {G\sigma_{u}^{2} } & 0 \\ 0 & {I\sigma_{e}^{2} } \\ \end{array} } \right]$$

where $\sigma_{u}^{2}$ and $\sigma_{e}^{2}$ are the polygenic and error variance components, **I** is the nxn identity matrix, and **G** is the n x n genomic relationship matrix. Univariate analyses were performed using the factored spectrally transformed linear mixed model (FaST-LMM^48^) software (C++ Version 2.07) that was available at github (https://fastlmm.github.io/). Apart from SNP p values, FaST-LMM automatically computed the SNP q values using the false-discovery rate (FDR^49^) correction method.

A bivariate linear mixed model was then applied to identify significant CP SNP associations with both traits. Specifically, the following bivariate linear mixed model was used:

$${\mathbf{Y}} = {\mathbf{WA}} + {\text{x}}\beta^{{\text{T}}} + {\mathbf{U}} + {\mathbf{E}}$$

with **U** ~ MN_nx2_(0, **G**, **V**_**g**_) and

$${\mathbf{E}}\sim {\text{MN}}_{{{\text{nx2}}}} \left( {0,{\mathbf{I}}_{{{\mathbf{nxn}}}} ,{\mathbf{V}}_{{\mathbf{e}}} } \right)$$

where **Y** is a *n* × 2 matrix of 2 phenotypes for n = 2586 animals, **W** is a n × 53 matrix of covariates (fixed effects) including hatch (36 classes) and mating group (17 classes); **A** is a *c* × 53 matrix of the corresponding coefficients including the intercept; *x* is a *n*-vector of marker genotypes (coded as 0, 1, and 2 according to the number of copies of the minor allele), *β* is 2-vector of marker effect sizes for the 2 phenotypes; **U** is a n × 2 matrix of random effects and **E** is a n × 2 matrix of random residuals. Furthermore, **G** is the n x n genomic relationship matrix (estimated as centered genomic matrix^7,50^), **V**_**g**_ is a 2 × 2 symmetric matrix of genetic (co)variance, I is a n × n identity matrix, **V**_**e**_ is a 2 × 2 symmetric positive definite matrix of residual variance component and MN_n×2_(0, **V**_**1**_, **V**_**2**_) denotes the n × 2 matrix normal distribution with mean 0, row covariance matrix **V**_**1**_ (n × n) and column covariance matrix **V**_**2**_ (2 × 2).

Association of each SNP with both traits was assessed by testing the null hypothesis that the marker effect sizes for both phenotypes are zero i.e. H_0_: β = 0, where β is a vector of the two marker effects, against the general alternative hypothesis H_1_: β ≠ 0. The Wald test statistic was used to infer the significant CP SNP associations. The genetic correlation (r_g_) was also estimated between the two traits. Bivariate analysis was performed using the GEMMA^51^ software (version 0.98.1).

For each association analysis, the estimation of the genomic inflation factor (*λ*) was used to assess potential systematic bias due to population structure or the analytical approach^52^. If the *λ* value was greater than 1, it provided evidence for some systematic bias^52^. If the *λ* value was less than or equal to 1, no adjustment was needed^53^. *λ* was estimated using the SNP & Variation Suite software (http://www.goldenhelix.com).

### Multiple-testing correction

For univariate association analyses, FaST-LMM corrected SNP p values for multiple comparisons, so there was no need for an additional correction, however, for bivariate analysis the FDR^49^ correction method was applied using R (http://www.r-project.org/). For all analyses, SNPs with FDR p values lower than 0.10 were considered as genome-wide significant.

### Selection of LD-independent SNPs

Results obtained from univariate and bivariate analyses were further subject to stepwise conditional and joint (cojo) analysis using the ‘*cojo-slct’* option and the GCTA^54^ tool to select the independent SNPs. The cojo-GCTA analysis corrects *β* and *p* values of neighboring SNPs (in a sliding window of 10 Mb) based on the LD between the SNPs. This ensures that the SNP with the lowest *p* value is selected first for conditioning the effect on neighboring loci based on the LD between the neighboring SNPs and the selected SNP. Following LD-based correction of effect, all SNPs that remained significant under a p value threshold (4.2e−06) are run through the same process in a stepwise manner. A p value threshold as high as 4.2e−06 (i.e. 1/total number of analyzed SNPs) was used here to declare the independent significant SNPs. In short, cojo analysis identifies: (1) the number of independent SNP signals in a region and (2) association signals due to the joint effect of several SNPs. To identify the independent CP associations, we used as input in the cojo-GCTA analysis the summary-level statistics obtained by the bivariate analysis. Specifically, the *b* estimates along with their standard errors were used to estimate t values for the SNPs and t values were finally converted to p values using R code (http://www.r-project.org/).

### Effect prediction of the independent CP significant SNPs and detection of positional candidate genes and published QTLs

To predict the consequences of the independent CP significant SNPs on genes, transcripts, protein sequence and regulatory regions, the Variant Effect Predictor (VEP, https://www.ensembl.org/Tools/VEP^55^) tool was employed with the latest release (Ensembl release 102, accessed: 18 December 2020).

Physical positions of SNPs were also obtained by the VEP tool using the GRCg6a assembly (https://www.ensembl.org/Gallus_gallus/Info/Annotation, GenBank Assembly ID: GCA_000002315.5, accessed: 18 December 2020). The VEP tool was also used to search for positional candidate genes and for published QTLs including the independent CP significant SNPs. Note that both Ensembl and NCBI RefSeq transcript databases were used. With regard to published QTLs, VEP retrieves information via connections with Animal QTL database (Animal QTLdb) and Online Mendelian Inheritance in Animals (OMIA) database for *Gallus gallus*.

### Bioinformatics analyses

We conducted ontological and functional analysis of the positional candidate genes in efforts to elucidate their functional role and relevance to the traits under study. First, modular enrichment analysis (MEA) using GeneCodis 4.0 (https://genecodis.genyo.es/^56,57^) was carried out. MEA removes the redundant terms and produces genes and annotations grouped in modules (or metagroups) which are functionally coherent and are ranked by their significance and relevance^57^. For MEA, we selected the species of *Gallus gallus* for the input genes and searched for Gene Ontology (GO) biological processes (BPs) as well as KEGG pathway significantly enriched concurrent annotations. Here, concurrent annotations with FDR p value lower than 0.05 were considered as significantly enriched.

Second, the LAGO tool (https://go.princeton.edu/cgi-bin/LAGO^58^) was used to infer the GO BP terms of the candidate genes. Since there were unknown genes during exploration of chicken GO annotations, the human GO annotations were used here. Computation of p values was based on the hypergeometric distribution and a p value cut-off equal to 0.10 was set as denoting significantly enriched terms. Candidate genes associated with enriched GO BP and pathways relevant to growth and reproduction processes were considered as functionally relevant to the traits under study and were thus nominated as candidate pleiotropic genes. Finally, the GO term Mapper (https://go.princeton.edu/cgi-bin/GOTermMapper) was employed to infer the GO slim molecular function terms of the pleiotropic candidate genes using human genes as input.

### Ethical approval

All animals included in this study were not subjected to any invasive procedures.

## Supplementary Information

Supplementary Table S1.

Supplementary Table S2.

Supplementary Table S3.

Supplementary Table S4.

Supplementary Figure S1.

## Data Availability

The data that support the findings of this study are available from Aviagen but restrictions apply to the availability of these data, which were used under license for the current study, and so are not publicly available. Data are however available from the authors upon reasonable request and with permission of Aviagen.
