# Supplementary material for: Detection of loci exhibiting pleiotropic effects on body weight and egg number in female broilers
Source: Sci Rep. 2021 Apr 2;11:7441. doi: 10.1038/s41598-021-86817-8 (PMC8018976; doi:10.1038/s41598-021-86817-8)
Supplement: Supplementary file 1 — Supplementary Table S1. [file 41598_2021_86817_MOESM1_ESM.pdf]

Detection of loci exhibiting pleiotropic effects on body weight and egg number in female broilers

Eirini Tarsani[1*], Andreas Kranis[2,3], Gerasimos Maniatis[2], Ariadne L. Hager-Theodorides[1], Antonios Kominakis[1]

[1]Department of Animal Science and Aquaculture, Agricultural University of Athens, Iera Odos 75, 11855, Athens, Greece

[2]Aviagen, Newbridge, Midlothian EH28 8SZ, UK

[3] The Roslin Institute, University of Edinburgh, EH25 9RG, Midlothian, United Kingdom

*corresponding author: etarsani@aua.gr

Table S1: Independent significant SNPs for body weight (BW), egg number (EN) and for both traits in broilers.

SNP ID	GGA	Position (bp)[1]	P-value from univariate or bivariate analysis	FDR p-value	P-value from COJO[2]	Trait(s)
rs14829026	1	56635594	6.03E-12	6.74E-09	1.47E-77	BW
rs13965175	1	163858807	2.58E-11	2.57E-08	1.22E-31	
rs315835465	1	191914153	1.17E-04	4.63E-02	2.07E-75	
rs13543487	2	27103453	1.59E-18	6.81E-15	2.22E-08	
rs317435075	2	96676941	1.68E-08	1.18E-05	2.88E-27	
rs15140482	2	107231066	5.21E-18	1.92E-14	4.68E-18	
rs15156742	2	133305723	3.11E-22	4.31E-18	2.66E-22	
rs317321451	3	38158316	8.03E-13	1.03E-09	1.87E-36	
rs314958778	3	52121842	1.07E-12	1.34E-09	1.09E-12	
rs313208295	4	80292623	8.62E-18	3.08E-14	1.48E-14	
rs14512409	5	9351944	3.94E-15	6.74E-12	1.83E-07	
rs314712068	6	35135780	1.37E-16	3.33E-13	2.35E-29	
rs313879964	7	36286374	2.09E-29	1.23E-24	7.69E-94	
rs316232445	9	22148850	8.18E-17	2.17E-13	3.02E-28	
rs313298834	12	18995645	1.29E-04	5.00E-02	1.07E-18	

rs312555087	13	18545613	7.72E-11	7.43E-08	9.19E-220	
rs314215039	15	12856857	4.64E-19	2.49E-15	9.84E-33	
rs313536194	19	9937564	3.41E-17	1.00E-13	3.46E-24	
rs314507428	24	6149411	2.88E-08	1.99E-05	2.16E-09	
rs316091218	25	458122	1.36E-05	6.32E-03	1.21E-35	
rs316709322	28	5041307	3.14E-05	1.38E-02	1.35E-12	
rs314413172	2	129107513	3.24E-06	7.99E-02	3.37E-06	EN
rs314405198	3	33580904	3.27E-06	7.99E-02	3.39E-06	
rs313298834	12	18995645	1.62E-06	6.35E-02	1.68E-06	
rs313045367	26	362590	2.69E-06	7.99E-02	2.60E-06	
rs16212041	28	3892872	7.86E-07	6.35E-02	7.72E-07	
rs315275636	1	6206137	7.91E-12	2.70E-08	1.17E-12	BW and EN
rs317275973	1	23082139	1.82E-14	1.62E-10	1.29E-17	
rs312392044	1	35990963	1.41E-13	8.96E-10	2.71E-14	
rs316780156	1	87938572	8.75E-06	0.005942	2.93E-07	
rs315007062	1	100596308	3.22E-09	4.86E-06	1.74E-09	
rs315995534	1	110869322	8.56E-07	0.000739	2.13E-06	
rs317073055	1	121235094	4.67E-13	2.39E-09	7.06E-14	
rs317590244	1	136269771	2.57E-11	7.88E-08	3.78E-12	
rs316472061	1	185926511	1.10E-13	7.39E-10	1.95E-14	
rs14135719	2	8489508	2.87E-11	8.57E-08	4.41E-12	
rs13543487	2	27103453	1.92E-13	1.16E-09	5.89E-14	
rs317979230	2	59469333	1.19E-14	1.12E-10	3.06E-15	
rs315191969	2	75552665	2.04E-08	2.49E-05	8.15E-08	
rs15140482	2	107231066	2.23E-11	7.01E-08	3.28E-12	
rs15156742	2	133305723	1.42E-15	1.96E-11	3.03E-16	
rs313125064	3	21986853	2.93E-07	0.000271	2.13E-13	
rs317668107	3	33354124	1.45E-11	4.81E-08	2.22E-17	
rs314958778	3	52121842	1.58E-08	2.01E-05	2.04E-08	
rs313973628	4	8970286	1.48E-10	3.46E-07	1.88E-08	
rs313178030	4	26530662	2.27E-13	1.34E-09	3.38E-14	
rs317953448	4	43384266	7.08E-12	2.46E-08	1.02E-12	
rs15608447	4	66459916	9.09E-09	1.24E-05	3.66E-10	
rs313208295	4	80292623	4.60E-11	1.31E-07	6.62E-12	
rs312798022	5	8828819	3.11E-12	1.26E-08	4.08E-13	
rs313257959	5	30658287	7.37E-14	5.11E-10	1.39E-14	
rs314038572	5	50471323	5.00E-10	9.73E-07	7.49E-11	
rs314529054	6	21832302	1.78E-07	0.000172	3.12E-07	
rs314712068	6	35135780	2.74E-10	5.93E-07	2.44E-10	
rs313879964	7	36286374	4.11E-22	2.42E-17	2.15E-22	
rs314425715	8	770143	1.21E-08	1.59E-05	3.17E-09	
rs317902708	8	21684030	1.30E-10	3.15E-07	3.88E-10	
rs317315660	9	17942760	9.72E-07	0.000818	4.68E-12	

rs14952656	10	17996013	1.62E-10	3.67E-07	1.08E-29	
rs316546378	11	5124955	2.87E-05	0.016969	4.87E-07	
rs318098582	11	18407493	2.66E-21	1.05E-16	5.21E-22	
rs318048363	12	6154483	1.09E-10	2.75E-07	1.63E-11	
rs318032338	13	16259361	7.64E-09	1.07E-05	6.22E-07	
rs317631529	14	5738298	1.86E-14	1.62E-10	1.16E-11	
rs314778226	15	4845973	3.27E-14	2.57E-10	4.93E-15	
rs317370260	17	1629390	0.000148	0.061232	2.89E-08	
rs313997974	18	6177837	9.66E-10	1.74E-06	5.29E-13	
rs313536194	19	9937564	1.66E-10	3.73E-07	4.10E-15	
rs317414603	20	6729013	4.47E-23	3.51E-18	7.37E-24	
rs314420361	21	698421	1.31E-15	1.96E-11	3.75E-16	
rs317101069	23	3379059	2.23E-15	2.77E-11	4.59E-09	
rs14291881	24	150829	2.59E-19	7.63E-15	4.07E-20	
rs316343530	26	2854350	2.24E-06	0.001699	7.48E-13	
rs315329074	27	6920352	4.23E-25	9.97E-20	6.13E-32	
rs314496246	28	3661043	1.42E-15	1.96E-11	1.98E-11	

[1]Positions are based on GRCg6a assembly
[2]COJO stands for conditional and joint analysis
