# Supplementary material for: Detection of loci exhibiting pleiotropic effects on body weight and egg number in female broilers
Source: Sci Rep. 2021 Apr 2;11:7441. doi: 10.1038/s41598-021-86817-8 (PMC8018976; doi:10.1038/s41598-021-86817-8)
Supplement: Supplementary file 2 — Supplementary Table S2. [file 41598_2021_86817_MOESM2_ESM.pdf]

Detection of loci exhibiting pleiotropic effects on body weight and egg number in female broilers

Eirini Tarsani[1*], Andreas Kranis[2,3], Gerasimos Maniatis[2], Ariadne L. Hager-Theodorides[1], Antonios Kominakis[1]

[1]Department of Animal Science and Aquaculture, Agricultural University of Athens, Iera Odos 75, 11855, Athens, Greece

[2]Aviagen, Newbridge, Midlothian EH28 8SZ, UK

[3] The Roslin Institute, University of Edinburgh, EH25 9RG, Midlothian, United Kingdom

*corresponding author: etarsani@aua.gr

Table S2: Positional candidate genes and reported QTLs for the independent cross phenotype significant SNPs.

SNP	GGA	Position (bp)[1]	Consequence (variant)	Positional candidate gene (gene ID)[2]	Gene description (biotype)	Start position - end position of gene (bp)	QTL(s)
rs315275636	1	6,206,137	intron variant	*CELF2 (ENSGALG00000034590)*	*CUGBP Elav-like family member 2 (protein coding)*	6,143,111-6,513,417	none
rs317275973	1	23,082,139	intron variant	*PTPRZ1 (ENSGALG00000040114)*	*protein tyrosine phosphatase, receptor type Z1 (protein coding)*	23,044,093-23,176,470	Feather colour extended black (ID:157158)
rs312392044	1	35,990,963	intron variant	*PTPRB (ENSGALG00000010049)*	*protein tyrosine phosphatase,*	35,964,985-36,023,189	none

					receptor type B (protein coding)		
rs316780156	1	87,938,572	intron variant, non coding transcript variant	*LOC112530378 (112530378)*	*uncharacterized LOC112530378 (lncRNA)*	87,894,824-88,208,405	none
rs315007062	1	100,596,308	intergenic variant				none
rs315995534	1	110,869,322	non coding transcript exon variant	*LOC112530939 (112530939)*	*uncharacterized LOC112530939 (lncRNA)*	110,866,971-110,879,038	none
rs317073055	1	121,235,094	intron variant	*EIF1AX (ENSGALG00000016410)*	*eukaryotic translation initiation factor 1A, X-linked (protein coding)*	121,229,301-121,238,473	none
rs317590244	1	136,269,771	5 prime UTR variant	*LOC112531515 (112531515)*	*uncharacterized LOC112531515 (protein coding)*	136,266,311-136,274,265	none
rs316472061	1	185,926,511	intron variant,non coding transcript variant<br>intron variant,non coding transcript variant<br>intron variant,non coding transcript variant	*(ENSGALG00000047983)*<br>*(ENSGALG00000054022)*<br>*LOC107052371 (107052371)*	*novel gene (lncRNA)*<br>*novel gene (lncRNA)*<br>*uncharacterized LOC107052371 (lncRNA)*	185,849,508-185,992,637<br>185,912,084-185,975,486<br>185,906,858-185,939,575	Tibia length (ID: 135878), Feed intake (IDs: 64551, 64552,64553,64555),Dry matter intake (ID: 64554)
rs14135719	2	8,489,508	intron variant,non coding transcript variant	*LOC112531942 (112531942)*	*uncharacterized LOC112531942 (lncRNA)*	8,477,606-8,512,826	Feather crested head (ID: 127112), Feather colour extended

							black (ID: 157162)
rs13543487	2	27,103,453	intergenic variant				Feather crested head (ID: 127112),pH of digestive tract contents (ID: 96617),Feather colour extended black (ID: 157164)
rs317979230	2	59,469,333	intergenic variant				Feather crested head (ID: 127112),Wattles weight (ID: 127117)
rs315191969	2	75,552,665	intron variant	*LOC101749223 (101749223)*	*unconventional myosin-X-like (protein coding)*	75,494,741-75,646,604	Feather crested head (ID: 127112),Wattles weight (ID: 127117)
rs15140482	2	107,231,06 6	intron variant	*NOL4 (ENSGALG00000015198)*	*nucleolar protein 4 like (protein coding)*	107,062,555-107,248,585	Feather crested head (ID: 127112),Wattles weight (ID: 127117)
rs15156742	2	133,305,72 3	intergenic variant				Wattles weight (ID: 127117)
rs313125064	3	21,986,853	intron variant	*TMEM206 (421371)*	*transmembrane protein 206 (protein coding)*	21,967,280-22,050,297	Comb weight (ID: 127114)

rs317668107	3	33,354,124	intergenic variant				Comb weight (ID: 127114) ,Jejunum length (ID: 96631)
rs314958778	3	52,121,842	intergenic variant				Comb weight (ID: 127114), Residual feed intake (ID: 64556)
rs313973628	4	8,970,286	intergenic variant				none
rs313178030	4	26,530,662	intergenic variant				none
rs317953448	4	43,384,266	intergenic variant				Ileum weight (ID: 96634)
rs15608447	4	66,459,916	intron variant<br>intron variant, non coding transcript variant	*SLAIN2 (ENSGALG00000014115)*<br>*LOC107053243 (107053243)*	*SLAIN motif family member 2 (protein coding)*<br>*uncharacterized LOC107053243 (lncRNA)*	66,459,339-66,485,331<br>66,450,553-66,472,818	none
rs313208295	4	80,292,623	intron variant	*SORCS2 (ENSGALG00000015554)*	*sortilin related VPS10 domain containing receptor 2 (protein coding)*	79,986,601-80,511,445	none
rs312798022	5	8,828,819	intron variant,non coding transcript variant<br>intron variant,non coding transcript variant	*(ENSGALG00000051198)*<br>*LOC107053374 (107053374)*	*novel gene (lncRNA)*<br>*uncharacterized LOC107053374 (lncRNA)*	8,788,839-8,838,256<br>8,748,372-8,865,519	none
rs313257959	5	30,658,287	intron variant	*FMN1 (ENSGALG00000009723)*	*formin 1 (protein coding)*	30,598,320-30,751,690	none
rs314038572	5	50,471,323	intron variant	*MARK3 (ENSGALG00000011505)*	*microtubule affinity regulating kinase 3 (protein coding)*	50,439,883-50,500,972	none

rs314529054	6	21,832,302	intron variant	*CPEB3 (ENSGALG00000006912)*	*cytoplasmic polyadenylation element binding protein 3 (protein coding)*	21,812,767-21,889,131	Ovary weight (ID: 147744),Body weight 21 days (ID: 95406)
rs314712068	6	35,135,780	intron variant<br><br>non coding transcript exon variant	*EBF3 (ENSGALG00000010461)*<br><br>*LOC107053752 (107053752)*	*early B cell factor 3 (protein coding)*<br>*uncharacterized LOC107053752 (lncRNA)*	35,112,704-35,230,061<br><br>35,127,705-35,152,732	none
rs313879964	7	36,286,374	intron variant	*ACVR1 (ENSGALG00000037301)*	*activin A receptor type 1 (protein coding)*	36,257,915-36,304,135	none
rs314425715	8	770,143	intron variant,non coding transcript variant<br><br>intron variant	*(ENSGALG00000048836)*<br><br>*AMY2A (414140)*	*novel gene (lncRNA)*<br>*amylase, alpha 2A (pancreatic) (protein coding)*	715,737-835,787<br><br>440,098-801,038	Ileum weight (ID: 96639)
rs317902708	8	21,684,030	intron variant	*MAST2 (424606)*	*microtubule associated serine/threonine kinase 2 (protein coding)*	21,613,724-21,776,006	Feathered feet (ID: 127123),Body weight 21 days (ID: 95408)
rs317315660	9	17,942,760	intron variant,non coding transcript variant<br><br>intron variant	*(ENSGALG00000050743)*<br><br>*TBL1XR1 (426284)*	*novel gene (lncRNA)*<br>*transducin beta like 1 X-linked receptor 1 (protein coding)*	17,900,261-17,996,324<br><br>17,895,996-18,186,649	Wattles weight (ID: 127118)
rs14952656	10	17,996,013	intron variant	*CHSY1 (ENSGALG00000026468)*	*chondroitin sulfate synthase 1 (protein coding)*	17,984,884-18,044,858	none

rs316546378	11	5,124,955	intron variant,non coding transcript variant	*(ENSGALG00000053968)*	*novel gene (lncRNA)*	5,102,154-5,159,147	Feed intake (ID: 64559)
rs318098582	11	18,407,493	missense variant	*ZC3H18 (ENSGALG00000006118)*	*zinc finger CCCH-type containing 18 (protein coding)*	18,379,252-18,412,636	none
rs318048363	12	6,154,483	intron variant,non coding transcript variant	*LOC107054451 (107054451)*	*uncharacterized LOC107054451 (lncRNA)*	6,145,475-6,164,001	none
rs318032338	13	16,259,361	intergenic variant				Feathered feet (ID: 127125),Breast muscle weight (ID: 96643),Feed conversion ratio (ID: 64562),Feather colour extended black (ID: 157195)
rs317631529	14	5,738,298	intron variant	*CACNA1H (ENSGALG00000005215)*	*calcium voltage-gated channel subunit alpha1 H (protein coding)*	5,667,870-5,837,019	Wattles length (ID: 127121),Body weight 36 days (ID: 64519)
rs314778226	15	4,845,973	intergenic variant				none
rs317370260	17	1,629,390	intergenic variant				Egg production rate (ID: 24945)
rs313997974	18	6,177,837	intergenic variant				Breast muscle weight (ID: 96653)

rs313536194	19	9,937,564	intergenic variant				Abdominal fat percentage (ID: 71126),Dry matter digestibility (ID: 95424)
rs317414603	20	6,729,013	intergenic variant				none
rs314420361	21	698,421	intron variant	*NPHP4 (ENSGALG00000000966)*	*nephrocystin 4 (protein coding)*	693,159-714,164	Body weight (ID: 95430),Egg shell color (ID: 24964)
rs317101069	23	3,379,059	synonymous variant	*EXTL1 (100859367)*	*exostosin like glycosyltransferase 1 (protein coding)*	3,374,545-3,380,690	none
rs14291881	24	150,829	intron variant	*VPS11 (ENSGALG00000029536)*	*VPS11, CORVET/HOPS core subunit (protein coding)*	77,314-151,086	none
rs316343530	26	2,854,350	intron variant	*PLXNA2 (419857)*	*plexin A2 (protein coding)*	2,754,008-3,101,360	Excreta weight (ID: 96622),Tibia length (ID: 135881)
rs315329074	27	6,920,352	intron variant	*CACNB1 (777366)*	*calcium voltage-gated channel auxiliary subunit beta 1 (protein coding)*	6,913,935-6,926,259	Wattles weight (ID: 127120),Comb weight (ID: 127127),Femur bone mineral content (ID: 130479),Femur weight (ID: 130480),Proventriculus weight (ID: 96672),Body

							weight hatch (ID: 135726)
rs314496246	28	3,661,043	intergenic variant				Feather crested head (ID: 127113)

[1]Positions are based on GRCg6a assembly.

[2]Note that Ensembl and RefSeq transcript databases were used in VEP tool to identify genes. Gene IDs refer to Ensembl gene IDs ('ENSGAL_') or NCBI gene IDs (numerical).
