# Supplementary material for: Detection of loci exhibiting pleiotropic effects on body weight and egg number in female broilers
Source: Sci Rep. 2021 Apr 2;11:7441. doi: 10.1038/s41598-021-86817-8 (PMC8018976; doi:10.1038/s41598-021-86817-8)
Supplement: Supplementary file 3 — Supplementary Table S3. [file 41598_2021_86817_MOESM3_ESM.pdf]

# Detection of loci exhibiting pleiotropic effects on body weight and egg number in female broilers

Eirini Tarsani[1*], Andreas Kranis[2,3], Gerasimos Maniatis[2], Ariadne L. Hager-Theodorides[1], Antonios Kominakis[1]

[1]Department of Animal Science and Aquaculture, Agricultural University of Athens, Iera Odos 75, 11855, Athens, Greece

[2]Aviagen, Newbridge, Midlothian EH28 8SZ, UK

[3] The Roslin Institute, University of Edinburgh, EH25 9RG, Midlothian, United Kingdom

*corresponding author: etarsani@aua.gr

Table S3: Results by modular enrichment analysis.

| Concurrent annotations | Annotation ids | FDR p-value | Genes found |
|---|---|---|---|
| negative regulation of transcription by RNA polymerase II, Wnt signaling pathway, regulation of gene expression,positive regulation of transcription, DNA-templated, positive regulation of transcription by RNA polymerase II, regulation of transcription by RNA polymerase II, proteasome-mediated ubiquitin-dependent protein catabolic process, histone deacetylation, negative regulation of transcription, DNA-templated, response to dietary excess, lipid catabolic process, multicellular organism growth, white fat cell differentiation, adipose tissue development, fat pad development, | GO:0000122,gga04310,GO:0010468,GO:0045893,GO:0045944,GO:0006357,GO:0043161,GO:0016575,GO:0045892,GO:0002021,GO:0016042,GO:0035264,GO:0050872,GO:0060612,GO:0060613,GO:0090207,GO:0090263 | 0.004 | *TBL1XR1* |

| | | | |
|---|---|---|---|
| regulation of triglyceride metabolic process, positive regulation of canonical Wnt signaling pathway | | | |
| negative regulation of translation, regulation of alternative mRNA splicing, via spliceosome, mRNA processing, alternative mRNA splicing, via spliceosome, mRNA splice site selection | GO:0017148,GO:0000381,GO:0006397,GO:0000380,GO:0006376 | 0.004 | *CELF2* |
| protein dephosphorylation, dephosphorylation, peptidyl-tyrosine dephosphorylation, hematopoietic progenitor cell differentiation, axonogenesis, negative regulation of neuron apoptotic process, oligodendrocyte differentiation,learning or memory, regulation of myelination, regulation of oligodendrocyte progenitor proliferation | GO:0006470,GO:0016311,GO:0035335,GO:0002244,GO:0007409,GO:0043524,GO:0048709,GO:0007611,GO:0031641, GO:0070445 | 0.004 | *PTPRZ1* |
| intracellular protein transport, protein transport, autophagy, vesicle-mediated transport, Salmonella infection | GO:0006886,GO:0015031,GO:0006914,GO:0016192,gga05132 | 0.004 | *VPS11* |
| phosphorylation, protein phosphorylation, regulation of interleukin-12 production | GO:0016310,GO:0006468,GO:0032655 | 0.004 | *MAST2* |

| | | | |
|---|---|---|---|
| atrioventricular valve morphogenesis,heart development,positive regulation of transcription, DNA-templated,positive regulation of transcription by RNA polymerase II,positive regulation of osteoblast differentiation,Cytokine-cytokine receptor interaction,intracellular signal transduction,mesoderm formation,angiogenesis,phosphorylation,protein phosphorylation,determination of left/right symmetry,cell migration,G1/S transition of mitotic cell cycle,endocardial cushion morphogenesis,smooth muscle cell differentiation,positive regulation of BMP signaling pathway,TGF-beta signaling pathway,transforming growth factor beta receptor signaling pathway,peptidyl-threonine phosphorylation,positive regulation of cell migration,positive regulation of bone mineralization,regulation of ossification,BMP signaling pathway,neural crest cell migration,germ cell development,cellular response to growth factor stimulus,branching involved in blood vessel morphogenesis,positive regulation of alkaline phosphatase activity,ventricular septum morphogenesis,positive regulation of epithelial to mesenchymal transition,cellular response to BMP stimulus,gastrulation,negative regulation of signal transduction,dorsal/ventral pattern formation,negative regulation of extrinsic apoptotic signaling pathway,cardiac epithelial to mesenchymal transition,pharyngeal system development,gastrulation with mouth forming second,positive regulation of determination of dorsal identity,transmembrane receptor protein serine/threonine kinase signaling pathway,positive regulation of pathway-restricted SMAD protein phosphorylation,mesoderm development,endothelial cell activation,mesenchymal cell differentiation,negative regulation of activin receptor signaling pathway,activin receptor signaling pathway,BMP signaling pathway involved in heart development,mitral valve morphogenesis,pathway-restricted SMAD protein phosphorylation,endocardial cushion fusion,cardiac muscle | GO:0003181,GO:0007507,GO:0045893,GO:0045944,GO:0045669,gga04060,GO:0035556,GO:0001707,GO:0001525,GO:0016310,GO:0006468,GO:0007368,GO:0016477,GO:0000082,GO:0003203,GO:0051145,GO:0030513,gga04350,GO:0007179,GO:0018107,GO:0030335,GO:0030501,GO:0030278,GO:0030509,GO:0001755,GO:0007281,GO:0071363,GO:0001569,GO:0010694,GO:0060412,GO:0010718,GO:0071773,GO:0007369,GO:0009968,GO:0009953,GO:2001237,GO:0060317,GO:0060037,GO:0001702,GO:2000017,GO:0007178,GO:0010862,GO:0007498,GO:0042118,GO:0048762,GO:0032926,GO:0032924,GO:0061312,GO:0003183,GO:0060389,GO:0003274,GO:0060923,GO:0002526,GO:0003143,GO:0003289,GO:0061445,GO:1905007 | 0.004 | *ACVR1* |

| | | | |
|---|---|---|---|
| cell fate commitment,acute inflammatory response,embryonic heart tube morphogenesis,atrial septum primum morphogenesis,endocardial cushion cell fate commitment,positive regulation of epithelial to mesenchymal transition involved in endocardial cushion formation | | | |
| multicellular organism development,actin nucleation | GO:0007275,GO:0045010 | 0.004 | *FMN1* |

| | | | |
|---|---|---|---|
| Metabolic pathways,protein glycosylation,heparan sulfate proteoglycan biosynthetic process,Glycosaminoglycan biosynthesis - heparan sulfate / heparin | gga01100,GO:0006486,GO:0015012,gga00534 | 0.010 | *EXTL1* |
| Oocyte meiosis, regulation of translation, Progesterone-mediated oocyte maturation | gga04114,GO:0006417,gga04914 | 0.010 | *CPEB3* |
| Metabolic pathways, metabolic process, carbohydrate metabolic process,Starch and sucrose metabolism | gga01100,GO:0008152,GO:0005975,gga00500 | 0.015 | *AMY2A* |
| Metabolic pathways,Glycosaminoglycan biosynthesis - chondroitin sulfate / dermatan sulfate | gga01100,gga00532 | 0.025 | *CHSY1* |
| positive regulation of transcription, DNA-templated,positive regulation of transcription by RNA polymerase II | GO:0045893,GO:0045944 | 0.029 | *ACVR1,EBF3, TBL1XR1* |
| MAPK signaling pathway,Adrenergic signaling in cardiomyocytes,Cardiac muscle contraction | gga04010,gga04261,gga04260 | 0.031 | *CACNB1* |
| RNA transport,translation,translational initiation | gga03013,GO:0006412,GO:0006413 | 0.031 | *EIF1AX* |
| multicellular organism development,regulation of transcription, DNA-templated,positive regulation of transcription, DNA-templated,positive regulation of transcription by RNA polymerase II | GO:0007275,GO:0006355,GO:0045893,GO:0045944 | 0.034 | *EBF3* |
| phosphorylation,protein phosphorylation | GO:0016310,GO:0006468 | 0.038 | *MAST2,ACVR1* |
| MAPK signaling pathway, Calcium signaling pathway | gga04010,gga04020 | 0.044 | *CACNA1H* |
