# Supplementary material for: Detection of loci exhibiting pleiotropic effects on body weight and egg number in female broilers
Source: Sci Rep. 2021 Apr 2;11:7441. doi: 10.1038/s41598-021-86817-8 (PMC8018976; doi:10.1038/s41598-021-86817-8)
Supplement: Supplementary file 4 — Supplementary Table S4. [file 41598_2021_86817_MOESM4_ESM.pdf]

Detection of loci exhibiting pleiotropic effects on body weight and egg number in female broilers

Eirini Tarsani[1*], Andreas Kranis[2,3], Gerasimos Maniatis[2], Ariadne L. Hager-Theodorides[1], Antonios Kominakis[1]

[1]Department of Animal Science and Aquaculture, Agricultural University of Athens, Iera Odos 75, 11855, Athens, Greece

[2]Aviagen, Newbridge, Midlothian EH28 8SZ, UK

[3] The Roslin Institute, University of Edinburgh, EH25 9RG, Midlothian, United Kingdom

*corresponding author: etarsani@aua.gr

Table S4: Gene Ontology biological processes.

GO ID	GO BP	P-value	Number of genes	Genes found
GO:0060037	pharyngeal system development	0.0004	2	*ACVR1, PLXNA2*
GO:1901890	positive regulation of cell junction assembly	0.0008	2	*FMN1, NPHP4*
GO:0035329	hippo signaling	0.0012	2	*MARK3, NPHP4*
GO:0008016	regulation of heart contraction	0.0031	3	*CACNA1H, CACNB1, CELF2*
GO:0051130	positive regulation of cellular component organization	0.0037	6	*CPEB3, FMN1, NPHP4, PLXNA2, SLAIN2, VPS11*
GO:0060047	heart contraction	0.0043	3	*CACNA1H, CACNB1, CELF2*

GO:0003015	heart process	0.0048	3	*CACNA1H, CACNB1, CELF2*
GO:1903522	regulation of blood circulation	0.0050	3	*CACNA1H, CACNB1, CELF2*
GO:1901888	regulation of cell junction assembly	0.0060	2	*FMN1, NPHP4*
GO:0032501	multicellular organismal process	0.0060	16	*ACVR1, AMY2A, CACNA1H, CACNB1, CELF2, CHSY1, CPEB3, EBF3, EXTL1, FMN1, MAST2, NPHP4, PLXNA2, PTPRB, PTPRZ1, TBL1XR1*
GO:0035335	peptidyl-tyrosine dephosphorylation	0.0068	2	*PTPRB, PTPRZ1*
GO:0003002	regionalization	0.0088	3	*ACVR1, CHSY1, PLXNA2*
GO:0048646	anatomical structure formation involved in morphogenesis	0.0092	5	*ACVR1, CACNA1H, FMN1, PLXNA2, PTPRB*
GO:0051239	regulation of multicellular organismal process	0.0094	9	*ACVR1, CACNA1H, CACNB1, CELF2, CHSY1, CPEB3, MAST2, PLXNA2, PTPRZ1*
GO:0032273	positive regulation of protein polymerization	0.0122	2	*FMN1, SLAIN2*
GO:0035107	appendage morphogenesis	0.0141	2	*FMN1, PLXNA2*
GO:0035108	limb morphogenesis	0.0141	2	*FMN1, PLXNA2*
GO:0050684	regulation of mRNA processing	0.0147	2	*CELF2, CPEB3*
GO:0007275	multicellular organism development	0.0163	12	*ACVR1, CACNA1H, CHSY1, CPEB3, EBF3, EXTL1, FMN1, NPHP4, PLXNA2, PTPRB, PTPRZ1, TBL1XR1*
GO:0048522	positive regulation of cellular process	0.0166	12	*ACVR1, CACNA1H, CHSY1, CPEB3, EBF3, FMN1, NPHP4, PLXNA2, PTPRZ1, SLAIN2, TBL1XR1, VPS11*
GO:0007389	pattern specification process	0.0175	3	*ACVR1, CHSY1, PLXNA2*
GO:0140056	organelle localization by membrane tethering	0.0181	2	*NPHP4, VPS11*
GO:0050769	positive regulation of neurogenesis	0.0191	3	*CPEB3, PLXNA2, PTPRZ1*

GO:0048731	system development	0.0195	11	*ACVR1, CACNA1H, CHSY1, CPEB3, EXTL1, FMN1, NPHP4, PLXNA2, PTPRB, PTPRZ1, TBL1XR1*
GO:0022406	membrane docking	0.0199	2	*NPHP4, VPS11*
GO:0048736	appendage development	0.0201	2	*FMN1, PLXNA2*
GO:0060173	limb development	0.0201	2	*FMN1, PLXNA2*
GO:0043254	regulation of protein complex assembly	0.0214	3	*FMN1, SLAIN2, VPS11*
GO:0032502	developmental process	0.0219	13	*ACVR1, CACNA1H, CHSY1, CPEB3, EBF3, EXTL1, FMN1, MAST2, NPHP4, PLXNA2, PTPRB, PTPRZ1, TBL1XR1*
GO:0048167	regulation of synaptic plasticity	0.0221	2	*CPEB3, SORCS2*
GO:0030278	regulation of ossification	0.0236	2	*ACVR1, CHSY1*
GO:0001501	skeletal system development	0.0238	3	*CHSY1, EXTL1, FMN1*
GO:0007010	cytoskeleton organization	0.0244	5	*FMN1, MARK3, MAST2, NPHP4, SLAIN2*
GO:0035637	multicellular organismal signaling	0.0245	2	*CACNA1H, CACNB1*
GO:0008015	blood circulation	0.0258	3	*CACNA1H, CACNB1, CELF2*
GO:1902905	positive regulation of supramolecular fiber organization	0.0261	2	*FMN1, SLAIN2*
GO:0051962	positive regulation of nervous system development	0.0269	3	*CPEB3, PLXNA2, PTPRZ1*
GO:0003013	circulatory system process	0.0272	3	*CACNA1H, CACNB1, CELF2*
GO:0010720	positive regulation of cell development	0.0282	3	*CPEB3, PLXNA2, PTPRZ1*
GO:0044087	regulation of cellular component biogenesis	0.0291	4	*FMN1, NPHP4, SLAIN2, VPS11*
GO:0044089	positive regulation of cellular component biogenesis	0.0294	3	*FMN1, NPHP4, SLAIN2*
GO:0045597	positive regulation of cell differentiation	0.0299	4	*ACVR1, CPEB3, PLXNA2, PTPRZ1*
GO:0048705	skeletal system morphogenesis	0.0309	2	*CHSY1, FMN1*

GO:0051495	positive regulation of cytoskeleton organization	0.0314	2	*FMN1, SLAIN2*
GO:0048856	anatomical structure development	0.0325	12	*ACVR1, CACNA1H, CHSY1, CPEB3, EBF3, EXTL1, FMN1, NPHP4, PLXNA2, PTPRB, PTPRZ1, TBL1XR1*
GO:0032271	regulation of protein polymerization	0.0327	2	*FMN1, SLAIN2*
GO:0023052	signaling	0.0336	13	*ACVR1, CACNA1H, CACNB1, CHSY1, CPEB3, EXTL1, MARK3, MAST2, NPHP4, PLXNA2, PTPRZ1, SORCS2, TBL1XR1*
GO:0044057	regulation of system process	0.0341	3	*CACNA1H, CACNB1, CELF2*
GO:0007154	cell communication	0.0345	13	*ACVR1, CACNA1H, CACNB1, CHSY1, CPEB3, EXTL1, MARK3, MAST2, NPHP4, PLXNA2, PTPRZ1, SORCS2, TBL1XR1*
GO:0035295	tube development	0.0350	4	*ACVR1, FMN1, PLXNA2, PTPRB*
GO:0007610	behavior	0.0361	3	*CPEB3, NPHP4, PTPRZ1*
GO:0009653	anatomical structure morphogenesis	0.0373	7	*ACVR1, CACNA1H, CHSY1, FMN1, PLXNA2, PTPRB, PTPRZ1*
GO:0034329	cell junction assembly	0.0388	2	*FMN1, NPHP4*
GO:0061448	connective tissue development	0.0391	2	*CHSY1, TBL1XR1*
GO:0007611	learning or memory	0.0391	2	*CPEB3, PTPRZ1*
GO:0050789	regulation of biological process	0.0405	19	*ACVR1, CACNA1H, CACNB1, CELF2, CHSY1, CPEB3, EBF3, EXTL1, FMN1, MARK3, MAST2, NPHP4, PLXNA2, PTPRB, PTPRZ1, SLAIN2, SORCS2, TBL1XR1, VPS11*
GO:0010638	positive regulation of organelle organization	0.0410	3	*FMN1, SLAIN2, VPS11*

GO:0048468	cell development	0.0413	6	*ACVR1, CHSY1, CPEB3, NPHP4, PLXNA2, PTPRZ1*
GO:0043009	chordate embryonic development	0.0424	3	*ACVR1, PLXNA2, TBL1XR1*
GO:0051146	striated muscle cell differentiation	0.0430	2	*ACVR1, CACNA1H*
GO:0009792	embryo development ending in birth or egg hatching	0.0454	3	*ACVR1, PLXNA2, TBL1XR1*
GO:0048666	neuron development	0.0458	4	*CPEB3, NPHP4, PLXNA2, PTPRZ1*
GO:0010976	positive regulation of neuron projection development	0.0464	2	*CPEB3, PLXNA2*
GO:0048518	positive regulation of biological process	0.0468	12	*ACVR1, CACNA1H, CHSY1, CPEB3, EBF3, FMN1, NPHP4, PLXNA2, PTPRZ1, SLAIN2, TBL1XR1, VPS11*
GO:0060828	regulation of canonical Wnt signaling pathway	0.0473	2	*NPHP4, TBL1XR1*
GO:0007267	cell-cell signaling	0.0479	5	*CACNB1, CPEB3, NPHP4, SORCS2, TBL1XR1*
GO:0050794	regulation of cellular process	0.0481	18	*ACVR1, CACNA1H, CELF2, CHSY1, CPEB3, EBF3, EXTL1, FMN1, MARK3, MAST2, NPHP4, PLXNA2, PTPRB, PTPRZ1, SLAIN2, SORCS2, TBL1XR1, VPS11*
GO:0031334	positive regulation of protein complex assembly	0.0482	2	*FMN1, SLAIN2*
GO:0034622	cellular protein-containing complex assembly	0.0482	4	*CELF2, FMN1, SLAIN2, VPS11*
GO:0050890	cognition	0.0512	2	*CPEB3, PTPRZ1*
GO:0051258	protein polymerization	0.0521	2	*FMN1, SLAIN2*
GO:0070588	calcium ion transmembrane transport	0.0524	2	*CACNA1H, CACNB1*
GO:0018105	peptidyl-serine phosphorylation	0.0537	2	*MARK3, MAST2*

GO:0030154	cell differentiation	0.0544	9	*ACVR1, CACNA1H, CHSY1, CPEB3, MAST2, NPHP4, PLXNA2, PTPRZ1, TBL1XR1*
GO:0007268	chemical synaptic transmission	0.0554	3	*CACNB1, CPEB3, SORCS2*
GO:0098916	anterograde trans-synaptic signaling	0.0554	3	*CACNB1, CPEB3, SORCS2*
GO:0034330	cell junction organization	0.0562	2	*FMN1, NPHP4*
GO:0048513	animal organ development	0.0564	8	*ACVR1, CACNA1H, CHSY1, FMN1, NPHP4, PLXNA2, PTPRZ1, TBL1XR1*
GO:0099537	trans-synaptic signaling	0.0570	3	*CACNB1, CPEB3, SORCS2*
GO:0051640	organelle localization	0.0575	3	*NPHP4, PLXNA2, VPS11*
GO:0099536	synaptic signaling	0.0587	3	*CACNB1, CPEB3, SORCS2*
GO:0060562	epithelial tube morphogenesis	0.0597	2	*ACVR1, FMN1*
GO:0006470	protein dephosphorylation	0.0600	2	*PTPRB, PTPRZ1*
GO:0060070	canonical Wnt signaling pathway	0.0613	2	*NPHP4, TBL1XR1*
GO:0018209	peptidyl-serine modification	0.0613	2	*MARK3, MAST2*
GO:0009101	glycoprotein biosynthetic process	0.0629	2	*CHSY1, EXTL1*
GO:1903311	regulation of mRNA metabolic process	0.0652	2	*CELF2, CPEB3*
GO:0016043	cellular component organization	0.0667	12	*CACNB1, CELF2, CPEB3, FMN1, MARK3, MAST2, NPHP4, PLXNA2, PTPRZ1, SLAIN2, TBL1XR1, VPS11*
GO:0042692	muscle cell differentiation	0.0679	2	*ACVR1, CACNA1H*
GO:0048869	cellular developmental process	0.0687	9	*ACVR1, CACNA1H, CHSY1, CPEB3, MAST2, NPHP4, PLXNA2, PTPRZ1, TBL1XR1*
GO:1902903	regulation of supramolecular fiber organization	0.0730	2	*FMN1, SLAIN2*
GO:0030111	regulation of Wnt signaling pathway	0.0730	2	*NPHP4, TBL1XR1*
GO:0022412	cellular process involved in reproduction in multicellular organism	0.0737	2	*ACVR1, MAST2*

GO:0045666	positive regulation of neuron differentiation	0.0744	2	*CPEB3, PLXNA2*
GO:0071495	cellular response to endogenous stimulus	0.0760	4	*ACVR1, CACNA1H, CACNB1, CPEB3*
GO:0019538	protein metabolic process	0.0772	11	*ACVR1, CHSY1, CPEB3, EIF1AX, EXTL1, MARK3, MAST2, PTPRB, PTPRZ1, TBL1XR1, VPS11*
GO:0050767	regulation of neurogenesis	0.0773	3	*CPEB3, PLXNA2, PTPRZ1*
GO:0001701	in utero embryonic development	0.0782	2	*ACVR1, TBL1XR1*
GO:0044267	cellular protein metabolic process	0.0787	10	*ACVR1, CPEB3, EIF1AX, EXTL1, MARK3, MAST2, PTPRB, PTPRZ1, TBL1XR1, VPS11*
GO:0031331	positive regulation of cellular catabolic process	0.0796	2	*CPEB3, VPS11*
GO:0031346	positive regulation of cell projection organization	0.0800	2	*CPEB3, PLXNA2*
GO:0010648	negative regulation of cell communication	0.0807	4	*ACVR1, MARK3, NPHP4, SORCS2*
GO:0035239	tube morphogenesis	0.0808	3	*ACVR1, FMN1, PTPRB*
GO:0051128	regulation of cellular component organization	0.0811	6	*CPEB3, FMN1, NPHP4, PLXNA2, SLAIN2, VPS11*
GO:0071840	cellular component organization or biogenesis	0.0811	12	*CACNB1, CELF2, CPEB3, FMN1, MARK3, MAST2, NPHP4, PLXNA2, PTPRZ1, SLAIN2, TBL1XR1, VPS11*
GO:0023057	negative regulation of signaling	0.0812	4	*ACVR1, MARK3, NPHP4, SORCS2*
GO:0065007	biological regulation	0.0819	19	*ACVR1, CACNA1H, CACNB1, CELF2, CHSY1, CPEB3, EBF3, EXTL1, FMN1, MARK3, MAST2, NPHP4, PLXNA2, PTPRB, PTPRZ1, SLAIN2, SORCS2, TBL1XR1, VPS11*
GO:0098660	inorganic ion transmembrane transport	0.0826	3	*CACNA1H, CACNB1, TMEM206*
GO:0001503	ossification	0.0828	2	*ACVR1, CHSY1*

GO:0051094	positive regulation of developmental process	0.0831	4	*ACVR1, CPEB3, PLXNA2, PTPRZ1*
GO:0030182	neuron differentiation	0.0848	4	*CPEB3, NPHP4, PLXNA2, PTPRZ1*
GO:0019953	sexual reproduction	0.0870	3	*ACVR1, CACNA1H, MAST2*
GO:0009100	glycoprotein metabolic process	0.0875	2	*CHSY1, EXTL1*
GO:0071310	cellular response to organic substance	0.0884	6	*ACVR1, CACNA1H, CACNB1, CPEB3, EXTL1, PTPRZ1*
GO:0006816	calcium ion transport	0.0923	2	*CACNA1H, CACNB1*
GO:0051716	cellular response to stimulus	0.0929	13	*ACVR1, CACNA1H, CACNB1, CHSY1, CPEB3, EXTL1, MARK3, MAST2, NPHP4, PLXNA2, PTPRZ1, SORCS2, TBL1XR1*
GO:0007155	cell adhesion	0.0953	4	*ACVR1, FMN1, NPHP4, PLXNA2*
GO:0022610	biological adhesion	0.0966	4	*ACVR1, FMN1, NPHP4, PLXNA2*
GO:0009888	tissue development	0.0971	5	*ACVR1, CHSY1, FMN1, PLXNA2, TBL1XR1*
GO:0006887	exocytosis	0.0972	3	*CACNA1H, PTPRB, VPS11*
