# Supplementary figures and images for: Detection of loci exhibiting pleiotropic effects on body weight and egg number in female broilers
Source: Sci Rep. 2021 Apr 2;11:7441. doi: 10.1038/s41598-021-86817-8 (PMC8018976; doi:10.1038/s41598-021-86817-8)

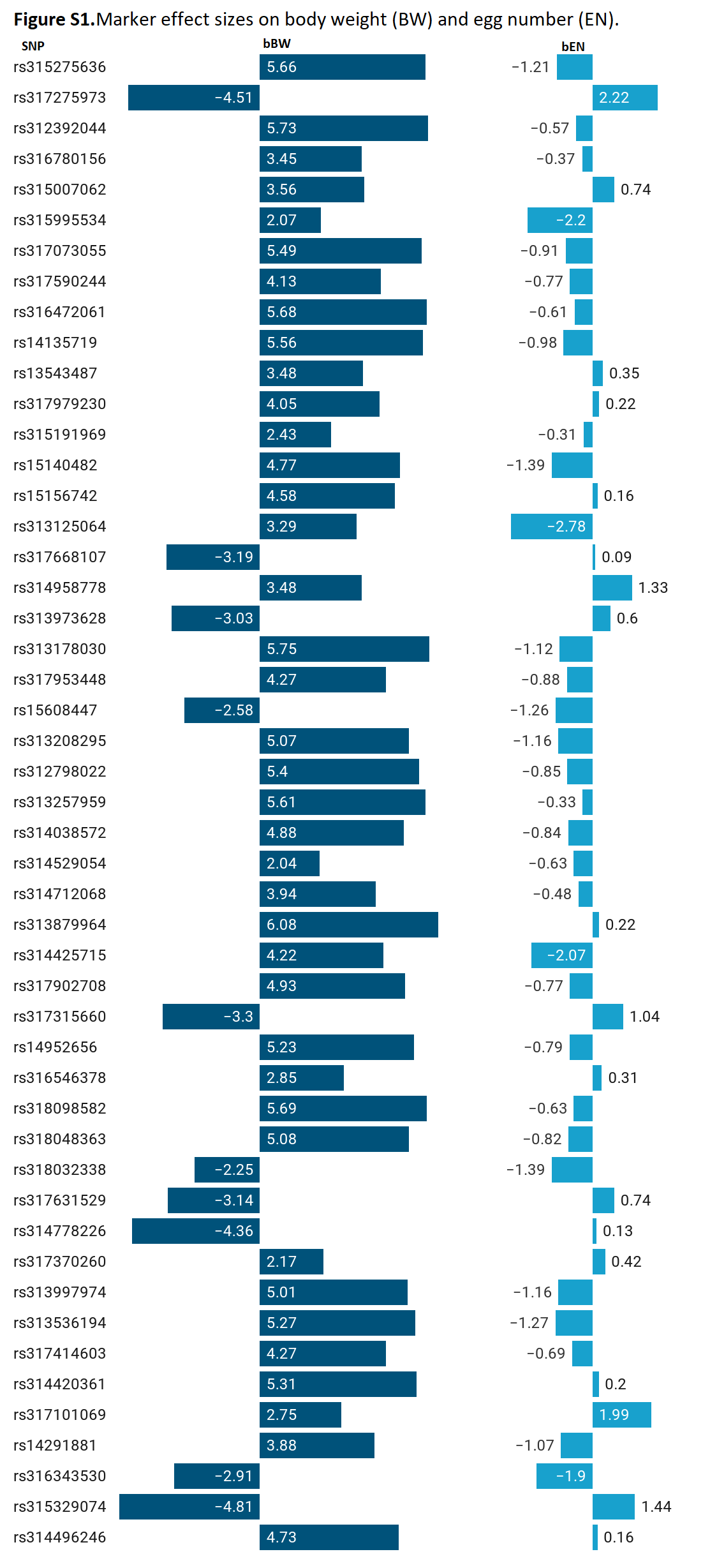

Supplement: Supplementary file 5 — Supplementary Figure S1. [file 41598_2021_86817_MOESM5_ESM.png]
